# Evaluation of Chemokines in the Gingival Crevicular Fluid of Children with Down Syndrome

**DOI:** 10.5005/jp-journals-10005-1528

**Published:** 2018-08-01

**Authors:** Veerakishore K Reddy, Naveen K Kommineni, Prathyusha Padakandla, Harshini Togaru, John P Indupalli, Swapna P Nanga

**Affiliations:** 1 Reader, Department of Pedodontics, CKS Teja Institute of Dental Sciences & Research, Tirupati, Andhra Pradesh, India; 2 Reader, Department of Pedodontics, CKS Teja Institute of Dental Sciences & Research, Tirupati, Andhra Pradesh, India; 3 Head, Department of Pedodontics, CKS Teja Institute of Dental Sciences & Research, Tirupati, Andhra Pradesh, India; 4 Senior Lecturer, Department of Pedodontics, CKS Teja Institute of Dental Sciences & Research, Tirupati, Andhra Pradesh, India; 5 Postgraduate Student, Department of Pedodontics, CKS Teja Institute of Dental Sciences & Research, Tirupati, Andhra Pradesh, India; 6 Assistant Professor, Department of Dental Surgery, Sri Venkateswara Institute of Medical Sciences, Tirupati, Andhra Pradesh, India

**Keywords:** Chemokines, Down syndrome, Gingival crevicu-lar fluid, Inflammation, Macrophage inflammatory protein 1a, Macrophage inflammatory protein 1p.

## Abstract

**Aim:**

The goal of the study was to detect the presence of macrophage inflammatory protein (MIP)-1α and MIP-1β and to estimate their levels in gingival crevicular fluid (GCF) of children with Down syndrome.

**Materials and methods:**

MIP-1α and MIP-1β levels were estimated in GCF samples of 20 healthy and 20 Down syndrome individuals. Gingival status was assessed by measuring the gingival index (GI), plaque index (PI), clinical attachment level (CAL), and probing pocket depth (PPD).

The GCF samples were obtained from the subjects and MIP-1α and MIP-1β levels were quantified by enzyme-linked immunosorbent assay (ELISA).

**Results:**

The mean MIP-1α concentrations in healthy and Down syndrome individuals were 209 and 1411 pg/μL respectively, and MIP-1α levels were 342 and 1404 pg/μL respectively.

The levels of MIP-1α and MIP-1β in the GCF of subjects with Down syndrome were significantly higher than in the healthy individual, and statistically significant differences were present among the two groups.

**Conclusion:**

The GCF showed dynamic changes according to the severity of periodontal disease, and the levels of MIP-1α and MIP-1β had a strong relationship with clinical parameters. The MIP-1α and MIP-1β can therefore be considered as novel biomarkers in the biological mechanism underlying the patho-genesis of periodontal disease.

**How to cite this article:** Reddy VK, Kommineni NK, Padakandla P, Togaru H, Indupalli JP, Nanga SP. Evaluation of Chemokines in the Gingival Crevicular Fluid of Children with Down Syndrome. Int J Clin Pediatr Dent 2018;11(4):288-293.

## INTRODUCTION

Down syndrome occurs when there is an extra copy of chromosome 21 and is characterized by the underdevelopment of mid-facial region, malocclusions, such as mandibular protrusion, open bite, and posterior crossbite as a consequence,^1^ and increased periodontal disease.

These individuals have more extensive gingival inflammation and earlier signs of alveolar bone loss, which is mainly localized around incisors in the lower front region.^2^ The prevalence of periodontal disease in Down syndrome persons varies depending on where they reside, with a higher prevalence in subjects residing in institutions as compared with those residing at home.^3^ Individuals with Down syndrome show colonization by various microorganisms that are in association with periodontal disease observed in early childhood.

The resulting altered composition of the subgingival plaque may lead to early initiation of periodontal disease.^4^ Of the inflammatory mediators present in diseased periodontium, chemokines, a family of chemotactic cytokines, have been involved in periodontal disease pathogenesis.^5^ Chemokines are critical mediators of cell migration and recruitment of their specific leukocytes to the sites of infection during immune surveillance, inflammation, and development.^6^ Macrophage inflammatory protein 1a is a cysteine-cysteine (CC) chemokine that was first identified in a lipopolysaccharide (LPS)-treated monocytic cell line.

It attracts monocytes, T lymphocytes, natural killer cells, dendritic cells, and granulocytes at inflammatory sites. The MIP-1α expression is increased in a number of diseases that are characterized by inflammation-induced bone loss.^7^ The MIP-1β, which is also a CC chemokine, is the bountiful expressed chemokine in periodontium.

The MIP-1β was initially characterized as a che-moattractant for activated CD4+ cells and has shown to selectively attract Th1 *vs* Th2 and effector cells. This observant selectivity for Th1 cells most likely results from the preferential expression of the MIP-1β receptor (CCR5) on Th1 cells, and suggests a potential role in directing the host responses along the proinflammatory pathway by the MIP-1β. The periodontal pathogenic microorganisms *Porphyromonas endodontalis, P. gingivalis,* and *Prevotella intermedia* produce MIP-1α and MIP-1β by stimulated neutrophils.^8^ The MIP-1α and MIP-1β are abundantly expressed chemokines in tissues periodonti-tis, with an expression localized in the connective tissue below the pocket epithelium of inflamed gingival tissues. They are also involved in the migration of macrophages to periodontal tissues.^9^

While studies have been performed to clinically assess gingivitis using plaque and gingival indices after placement of bands in orthodontic subjects, no study has been done to evaluate the levels of chemokines in the GCF of subjects with Down syndrome. Therefore, this study was designed to assess the levels of MIP-1α and MIP-1β in these individuals.

## MATERIALS AND METHODS

### Sample Size

There were 20 subjects per age group, which gave a 90% power to detect a difference in MIP-1α and MIP-1β levels in all control and Down syndrome groups. This group size also allowed for a 0.05 level of significance to be achieved.

### Study Population

The study sample consisted of 20 healthy subjects and 20 subjects with Down syndrome (13-18 years of age) from different special care homes in and around Tirupati. Caretakers of all of the children participating in the study duly signed an informed consent form.

Plaque index (Silness and Loe), GI (Loe and Silness), Russel’s periodontal index, and decayed, missing, filled teeth (DMFT)/decayed, extracted, filled teeth (deft) (<3) scores were recorded for each subject to avoid bias in the results.

### Inclusion and Exclusion Criteria

Subjects 13 to 18 years of age were recruited because more plaque accumulation is observed in individuals older than 5 years of age since the permanent molars start to erupt.

Periodontitis is commonly seen in these subjects because of difficulty in maintaining the proper oral hygiene since they have low IQs. Since individuals with Down syndrome exhibit early tooth loss, subjects with more than 15 functional teeth were included in the study along with mild-to-moderate inflammation with PPD 3 mm and CAL 2 mm.

Subjects with other systemic diseases, like human immunodeficiency virus and bleeding disorders, were excluded since systemic inflammation was present and may lead to false-positive results. Children using antimicrobial mouth rinses were also excluded in order to prevent false-negative results.

### Gingival Crevicular Fluid Sampling

The subjects were seated comfortably in an upright position in a dental chair with well-illuminated examination area.

A sterile mouth mirror and a Goldman/Fox Williams periodontal probe was used to clinically examine the peri-odontal status. The area was isolated using cotton rolls to prevent saliva contamination and GCF was collected using a 1 to 3 μL calibrated volumetric microcapillary pipette (Sigma Aldrich Chemical Company, USA; Catalog No. p0549) at the entrance of the gingival sulcus, and by gently touching the marginal gingiva.

**Fig. 1:**
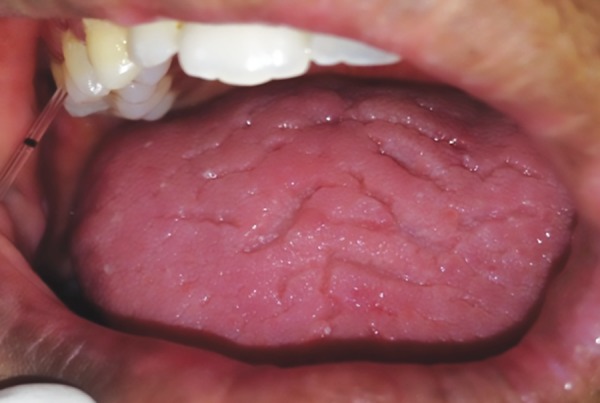
Gingival crevicular fluid collection from Down syndrome individual

By placing the tip of the pipette extracrevicularly (unstimulated) for 30 seconds, a standardized volume of 3 μL of GCF was collected for each test site using the markings on the micropipette from the buccal, lingual, or palatal sites of lower anteriors (most inflamed tissues) (Fig. 1).

### Analysis of MIP-1α and MIP-1β

**Fig. 2:**
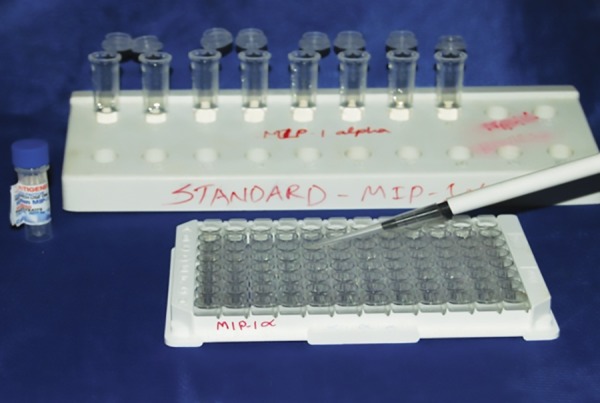
Addition of standard MIP-1α to GCF wells

**Fig. 3:**
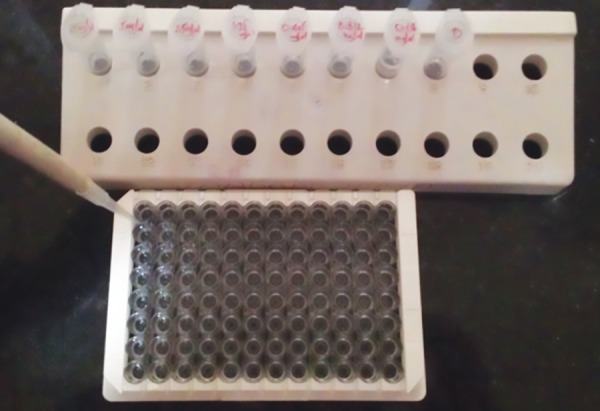
Addition of standard MIP-1β to GCF wells

In GCF, ELISA was performed using the quantitative sandwich enzyme immunoassay technique (Catalog Nos. DMP300 and DTM100; R and D Systems). Polyclonal antibodies specific for matrix metalloproteinase 3 and tissue inhibitor of metalloproteinase 1 were precoated onto a microplate. Standards and samples were pipetted into the wells, and any MIP-1α and MIP-1β was bound by the immobilized antibody (Figs 2 and 3).

After washing away any unbound substances, enzyme-linked polyclonal antibodies specific for MIP-1α and MIP-1β were added to the wells. Following a wash to remove any unbound antibody-enzyme reagent, a substrate solution was added to the wells. The color develops in proportion to the concentrations of total MIP-1α and MIP-1β (pro- and/or active) bound in the initial step.

**Fig. 4:**
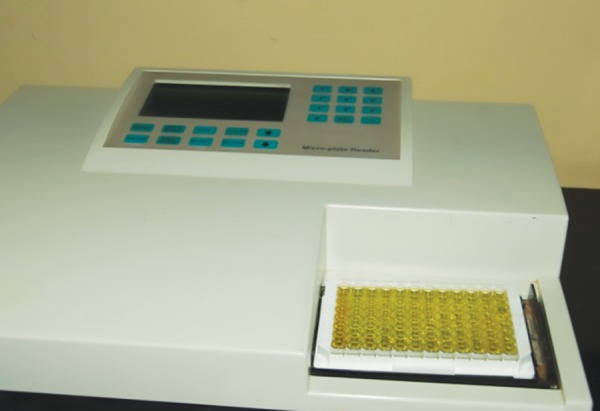
Enzyme-linked immunosorbent assay reader

After development was stopped, the color intensity was measured.

### Significance of the Methods used in This Study

The MIP-1α and MIP-1β concentrations were analyzed by ELISA (Fig. 4). In contrast, previous studies used filter paper strips and the Periastron 8000 and 6000.

This can result in nonspecific attachment of the analyte to filter paper fibers, which results in a false reduction in the detectable MIP-1α and MIP-1β levels, thus underestimating the correlation between their levels and tooth eruption.

### Statistical Analysis

The data were analyzed using Statistical Package for the Social Sciences (SPSS) program (version 11.5, SPSS Inc., Chicago, Illinois, USA) and unpaired t-test was applied for the analysis of the data.

## RESULTS

The data were analyzed using the SPSS program (version 11.5, SPSS Inc., Chicago, Illinois, USA). The data in Table 1 show that the mean PI of group I was 390 ± 0.152 pg/μL and in group II, it was 2198 ± 0.397 pg/μL.

The PI was higher in group II (2198 ± 0.397 pg/μL) (p < 0.001) than in group I (390 ± 0.152 pg/μL). Table 2 shows that the mean GI for group I was 312 ± 0.157 pg/μL and it was 2236 ± 0. 240 pg/μL for group II. The mean GI was higher in group II (2236 pg/μL), than in group I (312 pg/μL), and this difference was statistically significant (p < 0.001).

Table 3 shows that the mean PPD was 1.000 ± 0.000 pg/iL for group I and was 6.400 ± 1.046 pg/μL for group II. The mean PPD was higher for group II (6.400 pg/μL) than for group I (1.000 pg/μL), which was statistically significant (p < 0.001). Table 4 shows that the mean CAL for group I was 0000 ± 0.000 pg/μL and that for group II was 5300 ± 1.809 pg/μL.

**Table 1:** **Table 1:** Mean PI of groups I and II

*Groups*		*No. of samples*		*Mean (pg/μL)*		*SD*		*f-value*		*p-value*		*Significance*	
I		20		390		0.152		134.395		<0.001		S	
II		20		2198		0 397							

S: Significant; SD: Standard deviation

**Table 2:** **Table 2:** Mean GI for groups I and II

*Group*		*No. of samples*		*Mean (pg/μL)*		*SD*		*f-value*		*p-value*		*Significance*	
I		20		312		0.157		216.710		<0.001		S	
II		20		2236		0.240							

S: Significant; SD: Standard deviation

**Table 3:** **Table 3:** Mean PPD for groups I and II

*Group*		*No. of samples*		*Mean (pg/μL)*		*SD*		*f-value*		*p-value*		*Significance*	
I		20		1000		0.000		171.011		< 0.001		S	
II		20		6400		1.046							

S: Significant; SD: Standard deviation

**Table 4:** **Table 4:** Mean CAL for groups I and II

*Group*		*No. of samples*		*Mean (pg/μL)*		*SD*		*f-value*		*p-value*		*Significance*	
I		20		0		0.000		104.903		<0.001		S	
II		20		5300		1.809							

S: Significant; SD: Standard deviation

**Table 5:** **Table 5:** Mean GCF concentrations of MIP-1α for groups I and II

*Group*		*No. of samples*		*Mean (pg/μL)*		*SD*		*f-value*		*p-value*		*Significance*	
I		20		209		0.064		726.865		<0.001		S	
II		20		1481		0.141							

S: Significant; SD: Standard deviation

**Table 6:** **Table 6:** Mean GCF concentrations of MIP-1β for groups I and II

*Group*		*No. of samples*		*Mean (pg/μL)*		*SD*		*f-value*		*p-value*		*Significance*	
I		20		342		0.100		618.990		<0.001		S	
II		20		1404		0.088							

S: Significant; SD: Standard deviation

**Graph 1:**
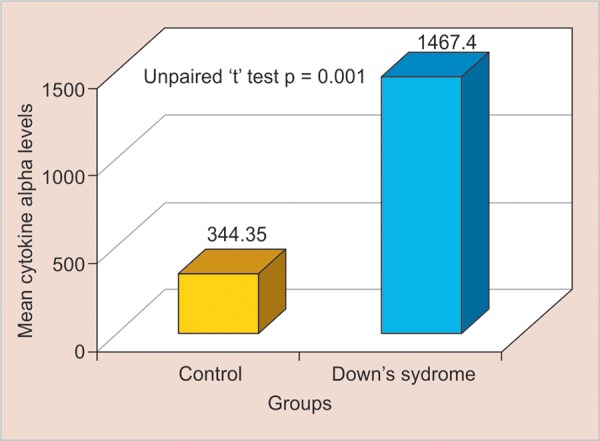
Test of significance for pairwise comparison of MIP-1α levels among subgroups

**Graph 2:**
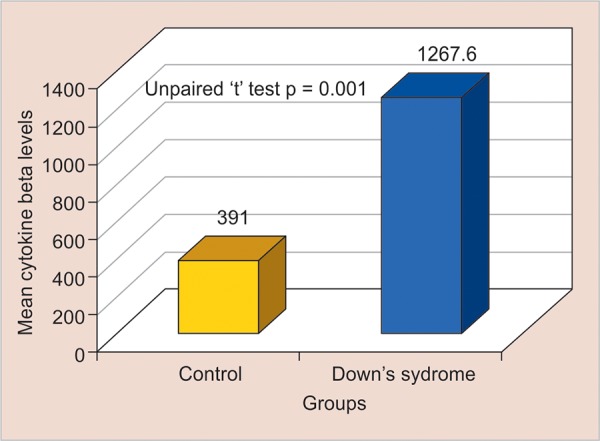
Test of significance for pairwise comparison of MIP-1β levels among subgroups

The mean CAL was higher in group II (5300 ± 1.809 pg/μL) than in group I (0000 ± 0.000 pg/μL), and this difference was statistically significant (p < 0.001). All of the samples for each group tested positive for MIP-1α and MIP-1β. The mean concentration of MIP-1α in GCF was 344.35 ± 31.75 pg/μL for group I and was 1467.40 ± 160.00 pg/μL for group II (Table 5 and Graph 1).

The mean concentration was notably higher in group II and the difference between these groups was statistically significant (p = 0.001). The mean concentration of MIP-1β in the GCF for group I was 391.00 ± 23.40 pg/μL and that for group II was 1267.60 ± 389.50 pg/μL

(Table 6 and Graph 2). The mean MIP-1β concentration for GCF was appreciably higher in group II than in group I, and this difference was statistically significant (p = 0.01).

## DISCUSSION

Periodontal disease is a chronic microbial and inflammatory condition distinctive by the presence of sulcular pathogenic bacteria, impaired host immune response, and destruction of the connective tissue attachments.^10^ Chemokines are the chemotactic cytokines that direct the recruitment and subsequent activation of specific leukocyte populations into inflamed periodontal tissues.^11^

Of the inflammatory mediators present in diseased peri-odontium, chemokines have been implicated in periodon-tal disease pathogenesis.^5^

Expression of MIP-1α in gingival tissue samples with chronic periodontal diseases has been investigated previously. Ryu and Choi^7^ recently reported that MIP-1α expression in gingival epithelial cells was induced by LPS, and they concluded that MIP-1α expression by gingival epithelial cells may be an important factor in initiating inflammation.

The ability of gingival epithelial cells to produce MIP-1α may provide a sustained source of this chemo-kine, thereby modulating the host response to inflammation in the gingival sulcus and in the surrounding gingival epithelium.^12^ Another chemokine, MIP-1β, also called CCL4, is considered to be appreciably expressed chemokine in periodontitis. Kabashima et al^13^ detected MIP-1β-producing cells in inflamed gingival samples collected from patients with chronic periodontitis.

The mean concentration of MIP-1α in GCF was found to be lower in group I (209 pg/μL) than in group II (1481 pg/μL). These levels increased proportionately from groups I to II and showed a positive correlation with clinical parameters. The possible reason for this increase in levels of MIP-1α in the GCF in this study may be the control of leukocyte migration depending on the combined actions of adhesion molecules and a large number of chemokines and their receptors.

When GCF MIP-1α concentrations in groups I and II were compared, the differences were statistically significant (p < 0.001), suggesting that MIP-1α concentrations in GCF increased actively from groups I to II. The MIP-1α levels increased proportionately from groups I to II, further confirming that MIP-1α was actively secreted by the predominant cells of periodontal disease activity.

The variability in MIP-1α and MIP-1β concentrations within subjects of each group could be attributed to their role in the different stages of disease progress at the time of GCF sample collection. The results of the present study agree with those reported by Gemmell et al^14^ who demonstrated that MIP-1α was expressed in the gingival tissues of subjects with mild-to-moderate periodontitis and that the levels correlated with the degree of inflammation. The results of our study contradict those of Emingil et al^15^ and Fokkema et al.^16^ Emingil et al^15^ reported that subjects with generalized aggressive periodontitis and those with chronic periodontitis have similar MIP-1α and MIP-1β levels in GCF samples when compared with gingivitis and periodontal healthy subjects.

They explained that the low MIP-1α levels in the periodontitis group could also be because of a lack of macrophages and subsets of lymphocytes with specific receptors for MIP-1α. Increased concentrations of MIP-1α and MIP-1β were detected in various systemic diseases, such as osteoarthritis,^17^ rheumatoid arthritis,^17^ congestive heart failure,^18^ multiple myeloma,^19^ and asthma.^20^ It was suggested that MIP-1α and MIP-1β were expressed by subchondral bone marrow stromal cells isolated from osteoarthritis and rheumatoid arthritis.^18^ The MIP-1α has been implicated in the pathogenesis of many diseases, and high levels of it in systemic circulation as a result of periodontal diseases may increase the risk for atherosclerosis and the other diseases mentioned above.

In the present study, the mean concentrations of MIP-1β in GCF were found to increase proportionately from healthy (342 pg/μL) to periodontitis individuals (Down syndrome group; 1404 pg/μL). The results of the present study are in agreement with those of Garlet et al,^21^ who advocated that MIP-1β was more prevalent and intensely expressed in patients with chronic periodontitis compared with the control subjects (p < 0.001). Mohamed et al^22^ demonstrated higher levels of IL-8 and MIP-1β in the GCF of subjects with diabetes. The results of the present study are contrary to those of Emingil et al^15^ and Fokkema et al.^16^ The former reported that patients with generalized aggressive and chronic periodontitis have a similar GCF MIP-1β levels when compared with gingivitis and periodontal healthy subjects.

They expressed that the low MIP-1β levels in the periodontitis group could also be because of a lack of macrophages and subsets of lymphocytes with specific receptors for MIP-1β. Fokkema et al^16^ reported that the levels of MIP-1β were similar between periodontitis and healthy subjects. Due to the lack of studies on the chemokine levels in individuals with Down syndrome in the pediatric dental research literature, we attempted to establish their role as diagnostic biomarkers in these subjects.

Our findings may help in the establishment of preventive measures to control the progression of the periodontal disease.

## CONCLUSION

Within the limitations of our study, the data indicate that MIP-1α and MIP-1β in GCF show dynamic changes according to the severity of periodontal disease, and their levels have a strong relationship with clinical parameters. Therefore, they can be used as markers of gingival inflammation.

However, further longitudinal studies are needed to determine the concentrations of MIP-1α and MIP-1β in periodontal disease tissues and GCF, to clarify their role in the periodontitis pathogenesis, and to validate MIP-1α and MIP-1β as novel biomarkers for periodontal disease progression.
